# Cost-Effective Control of Drug-Resistant TB: Listening to Other Voices

**DOI:** 10.1371/journal.pmed.0030542

**Published:** 2006-12-26

**Authors:** Jose Luis Portero, Maria Rubio

Drug-resistant tuberculosis is mainly a phenomenon caused by physicians, patients, and health-care systems through incorrect treatments, noncompliance, and poor delivery of tuberculosis services, respectively. These man-made facts could be avoided with appropriate actions. However, the weakness of health systems in the tuberculosis high-burden countries hampers successful control.

Public health priorities in resource-poor settings have marginalized tuberculosis cases resistant to first-line treatment. Nevertheless, patients have been claiming their rights to be treated despite their drug resistance pattern. Nowadays, tuberculosis programs try to address drug resistance issues. However, pilot experiences in low-resource settings do not fully answer to the real challenges in the field to scale up second-line drug treatments [1].

Governments from high-burden countries must enhance their commitments with their respective communities to provide better health and to alleviate poverty. Regarding drug-resistant tuberculosis control, these actions would improve organization and effectiveness in all levels of the tuberculosis programs to avoid misuse of resources. In addition, it is a must to address ignorance about tuberculosis transmission and treatment, social stigma, and discrimination. However, the current program design to control drug-resistant tuberculosis underestimates the environment of poverty suffered by the patients. In this sense, it is paradigmatic that one of the obstacles to following the treatment in the last pilot project published was that the patients were unable to buy symptomatic drugs to relieve the second-line drug side effects [2]. Governments, the World Health Organization, physicians, and technocrats must open their eyes to the reality in the field [3].

Unfortunately, we are far worldwide from a reliable system to fight drug-resistant tuberculosis. The complexity and requirements of treating resistant cases generally exceed the average available health care. In the other hand, the current cost of second-line anti-tuberculosis drugs is unbearable for the developing world and is a great obstacle to the scaling-up of the treatment. The cost of the drugs and the laboratory supplies is not appropriate for developing countries. No significant steps have been taken to put in practice a coordinated system to manage drug resistance in the community. Cost-effectiveness studies do not usually reflect the hidden costs for the patients and the real cost of the interventions in the present conditions. Feasibility measures do not take into account most of the socioeconomic barriers in the field.

There is a lack of independent opinions regarding drug-resistant tuberculosis, so the article by Resch et al. is very valuable [4]. Tuberculosis experts are in danger of listening only to their own words enclosed in a technocrat circle. It would be desirable to open tuberculosis control to civil society and to listen to other voices.
